# Impaired Balance in Patients with Fibromyalgia Syndrome: Predictors of the Impact of This Disorder and Balance Confidence

**DOI:** 10.3390/ijerph17093160

**Published:** 2020-05-01

**Authors:** Ana Peinado-Rubia, María C. Osuna-Pérez, Daniel Rodríguez-Almagro, Noelia Zagalaz-Anula, María C. López-Ruiz, Rafael Lomas-Vega

**Affiliations:** 1 AFIXA Fibromyalgia Association, Jaén, Spain; abpr0003@red.ujaen.es; 2 Department of Health Sciences, University of Jaén, 23071 Jaén, Spain; dralmagro4@gmail.com (D.R.-A.); nzagalaz@ujaen.es (N.Z.-A.); mlruiz@ujaen.es (M.C.L.-R.); rlomas@ujaen.es (R.L.-V.)

**Keywords:** fibromyalgia, vestibular diseases, postural balance, quality of life

## Abstract

Patients with fibromyalgia syndrome (FMS) have a nonspecific postural balance disorder and a greater prevalence of falls. Objective: to clarify which aspects of maintaining balance are associated with the impact of the disorder and with balance confidence. Methods: A total of 182 persons with FMS agreed to participate in this study. After re-evaluation, 57 fully met inclusion criteria: age 40–70 years and moderate-severe impact of the illness according to the Fibromyalgia Impact Questionnaire (FIQ). All participants underwent a posture control analysis with a stabilometric platform, an evaluation of the perception of verticality and an exploration of the vestibular system via functional tests. Additionally, they self-completed questionnaires about balance confidence, central sensitization, pain catastrophizing, kinesiophobia, dizziness and days with episodes of instability. Results: The FIQ was associated with central sensitization and dizziness, which explained 56% of its variance (AdjR2 = 0.566), while days with instability, kinesiophobia and dizziness also explained more than half of the variance of the balance confidence scale (AdjR2 = 0.527). A high percentage of positive responses was found for functional tests (>50%) and a high dispersion in the stabilometric parameters. Conclusion: the detection of factors susceptible to intervention, such as disability due to dizziness, takes on special relevance in patients with FMS.

## 1. Introduction

Fibromyalgia syndrome (FMS) is a disorder principally characterized by chronic generalized pain, with increased sensitivity to pain with a low pain threshold, which is normally accompanied by non-restorative sleep, tiredness, rigidity, instability, cognitive difficulties, and mood disorders [1]. FMS affects approximately 0.5%–5% of the global population, and notably has ambiguity in its diagnosis, uncertainty in the understanding of its physiopathology, and is difficult to treat [2].

Recent studies have shown that patients with FMS present with a nonspecific postural balance disorder and an increased prevalence of falls [3]. The magnitude of this balance disorder may be related with the increase in pain, loss of quality of life, and balance confidence [4,5]. Evidence shows a significant correlation between balance confidence (measured with the Activities-Specific Balance Confidence—ABC) and disease severity measured with the fibromyalgia impact questionnaire FIQ (r = −0.64) [3]. Patients with FMS identify balance issues as one of the ten most debilitating symptoms of this disease; with a reported prevalence of 45% [6].

The uncertainty of balance tests has hampered the finding of a characteristic dysfunctional pattern for this pathology. The scarce data are unclear, however, appear to suggest that these patients present with poor integration of visual and vestibular afferents with a sensorimotor preference when balancing, causing a deficient balance system [7,8,9]. They also present with an abnormally slow and irregular gait [10,11], lower scores in the assessment of balance confidence, and a higher average rate of falls than, for example, patients with rheumatoid arthritis (RA) or compared with healthy subjects. The incidence of falls in FMS patients has been reported as 1.75 falls/person each 6 months [3]. 

Falls prevention requires multiple rapid automatic corrections coordinated by the central nervous system; a process that appears to be affected in fibromyalgia [8]. No consensus exists on the predictive factors for falls, nevertheless, various studies have observed that the perception of postural instability and the deficit in the balance system performance could be considered as predictive factors [9,12,13]. 

Some studies have found an association between disability and worse scores on different balance assessments in patients with FMS [4,5,8]. Nevertheless, the studies that have analyzed the effectiveness of treatments based on balance in improving the clinical picture of these patients have not reached conclusive results [9]. 

This study aimed to clarify which variables, among functional balance tests, stabilometry and perception of verticality variables and central sensitization, pain catastrophizing, kinesiophobia and disability due to dizziness are related to the impact of the disease—the severity of symptoms measured with the fibromyalgia impact questionnaire (FIQ)—and with the confidence of these patients in their own stability measured with the activities-specific balance confidence (ABC) scale.

## 2. Materials and Methods 

### 2.1. Design and Sample

**Figure 1:**
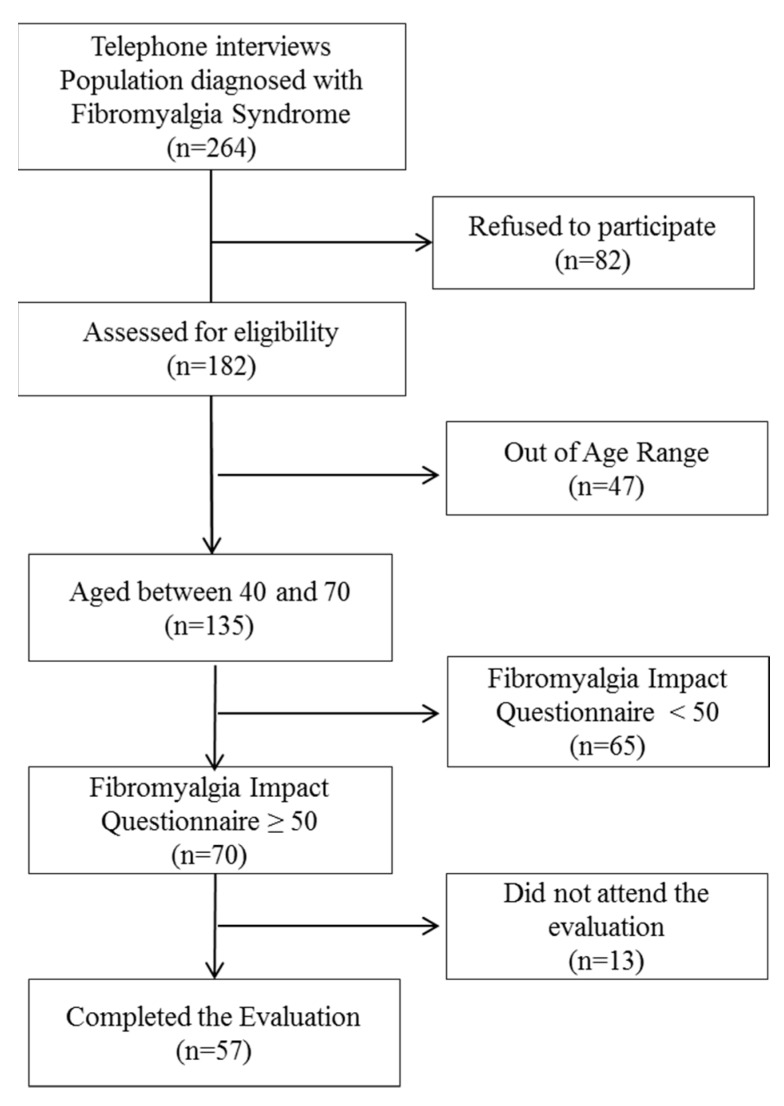
Flowchart showing the selection of participants.

An observational descriptive cross-sectional study was conducted in a sample of patients diagnosed with FMS who were recruited from the Fibromyalgia Association of Jaen city (AFIXA) from January to May 2019. Telephone contact was made with 264 possible participants who received detailed information regarding the study; of these, 182 agreed to participate. An in-person interview at the association was organized with each and they were re-evaluated to confirm that they met the inclusion criteria: aged 40–70 years, fully met the diagnostic criteria for fibromyalgia as described by the 2016 American College of Rheumatology (ACR), moderate to severe impact on quality of life, at the time of the interview, according to the categories described by the FIQ with a score equal to or greater than 50. Exclusion criteria were as follows: (a) cognitive impairment impacting ability to fill out the scales and questionnaires, (b) musculoskeletal surgical intervention in the preceding six months and/or acute traumatic pathology to the inferior limb(s), (c) musculoskeletal disease with deformity of the inferior limbs, (d) diagnosed with vestibular, visual and/or auditory pathology, and (e) neurologic illness that could be the cause of a balance disorder. A total of 70 participants met all the criteria, presenting with a moderate/severe limitation in quality of life at the time of inclusion, and a final 57 persons completed the whole study protocol. A flowchart showing the selection of participants in presented in Figure 1. 

The research protocol was approved by the Ethics committee of the University of Jaen and was designed and conducted in accordance with the Ethics code of the World Medical Association for studies including human participants (Declaration of Helsinki). All participants were provided with written informed consent to obtain voluntary agreement to participate in the study.

All participants were given a dossier of self-administered questionnaires. An examination of the vestibular system was performed based on a battery of functional tests, along with a stabilometric analysis, and an evaluation of the perception of verticality using a virtual reality system. Data collection was carried out on the premises of the University of Jaen.

### 2.2. Study and Instrument Variables

Demographic and anthropometric data such as sex, age, education level, occupation, civil status, height, weight, body mass index (BMI) were collected by well-trained interviewers.

### 2.3. Dependent Variables

The impact of the disease and disability of the symptoms was measured with the FIQ [14] and constituted one of the inclusion criteria. The FIQ is composed of 10 items that measure physical disability and the degree of specific symptoms such as pain, rigidity, fatigue, depression and anxiety, disability, and general well-being during the last week. Each symptom is measured on a response scale of 0 (absence of symptoms) to 10 (very severe). The FIQ total score ranges from 0 to 100, where higher values indicate a greater negative impact of the disease and can be classified into the following 3 categories: low impact (<50 points), moderate (50–75 points), and severe (>75 points). 

Balance confidence was recorded using thectivities-specific balance confidence scale (ABC-16) [15]. This is a 16-item questionnaire designed to identify a patient’s fear of falls through the degree of confidence with which patients are able to carry out specific everyday tasks without losing their balance. Each item can be scored from 0% (zero confidence) to 100% (complete confidence). The total score is between 0 and 100%, with higher values associated with higher balance confidence. Values lower than 67% have been shown to be both sensitive and specific for the prediction of falls, as well as for the considerable reduction in independence.

### 2.4. Predictor Variables

The variable “number of days with episodes of instability” was recorded by an experienced researcher, who asked participants to answer the following question: “How many days of the last week have you suffered episodes of instability?”, understanding these episodes, such as those situations where some of the following symptoms occur: sensation of movement or spinning (vertigo), feeling of lightheadedness or fainting (presyncope), sensation of insecurity when walking, feeling of floating or dizziness, changes in vision, blurred vision or disorientation. Response options range from zero (no day of the week) to seven (every day of the week). The conceptualization of episodes of instability was described by Horak [16].

The presence of central sensitization was measured with the central sensitization inventory (CSI) [17]. The CSI includes 25 items and evaluates a wide range of somatic and emotional symptoms common to central sensitization syndromes (CSS). The total score ranges from 0 to 100, where higher scores indicate greater severity of symptoms: a score of 40 has been established as the cut-off to determine the presence of central sensitization.

To assess pain catastrophizing the pain catastrophizing scale (PCS) was used [18]. The PCS is a self-administered scale that evaluates the patient’s catastrophic thoughts. It contains 13 items with a response scale from 0 (none/never) to 4 (all the time) and a total range of 0 to 52 points. Higher scores indicate a greater presence of catastrophic thoughts.

The fear of movement was measured with the Tampa scale for kinesiophobia (TSK) [19]. The TSK is a self-administered questionnaire with 11 items on fear of movement or of injury. The degree of agreement with each of the presented statements is recorded on a response scale from 1 (completely disagree) to 4 (completely agree), with a total range from 11 to 44 points. High scores indicate a greater fear of movement.

To evaluate disability due to vertigo the dizziness handicap inventory (DHI) was used [20]. This is a very useful multi-dimensional tool for quantifying self-perceived disability in patients with vertigo, dizziness, or instability, and its impact on activities of daily living. It contains 25 self-administered questions, with a total range of 0 to 100 points. A higher score indicates a greater degree of disability due to the vertiginous symptoms.

The perception of verticality was measured using the subjective visual vertical (SVV) test using a virtual reality device [21]. The SVV entails adjusting a randomly oriented line to a vertical position using virtual reality glasses. The initial orientation of the line is normally between 30 to 60 degrees to the right or left. Patients in a sitting and comfortable position have to align the line that appears on the screen to a vertical position without any external visual reference using a control that moves this line. The established normal values for the SVV are between –2.5° and 2.5° with respect to vertical [22].

To record static balance the Romberg test was done on a stabilometric platform with pressure resistant sensors (Sensor Medica. Roma, Italia) with a 400 x 400 mm surface and an acquisition frequency of 30Hz with FreeStep© Standard 3.0 software (Sensor Medica, Roma, Italy). Using this system, displacement of the center of pressure (CoP) is detected, similar to the center of gravity, in different situations of sensorial conflict (visual and somatosensory). This postural evaluation test was conducted using the same methodology used in other studies [23]. The patient is placed in a standing position on the platform. The feet should be bare and form a 30° angle, with 2 cm separation between the heels, and arms relaxed, extended and touching both sides of the body. Eyes should be looking horizontally at a fixed point on the wall, situated 2.5 m from the platform. The analysis period is 60 s, during which the patient is asked to remain relaxed and immobile, with a 60 s interval between each test. The following stabilometric parameters were recorded: mean velocity (v); root mean square in the medial lateral direction (RMSX) and in the anterior posterior direction (RMSY); X mean; Y mean. For each variable studied, 2 results were obtained corresponding to the measurements with open eyes (OE) and closed eyes (CE).

In terms of the functional evaluation of static and dynamic balance and neuromotor function, 7 function tests were conducted which assessed the function of the different structures and systems involved in postural control. These tests are classified in 3 analysis subscales: (a) standing balance/static balance: assessed with the Romberg test, sensitized Romberg test [24], and the Flamingo test or one-leg standing test [25]; (b) dynamic balance: evaluated with the Unterberger–Fukuda test [24] and its variation with cervical rotation to discriminate the nuchal reflex in postural tensions; (c) walking balance: assessed using the Babinski–Weils (blind gait) [26] and the tandem gait test [24]. 

### 2.5. Statistical Analysis

Continuous variables were described using means and standard deviations, whereas the categorical variables were described using frequencies and percentages. The Kolmogorov–Smirnov test was used to verify normal distribution of the continuous variables. 

The possible associations between different study variables were explored via bivariate analyses, using Pearson’s correlation for continuous variables and T-student for categorical variables. Variables with a significant association and showing a greater degree of association in the bivariate analysis were introduced stepwise into a multiple linear regression model for each of the study variables. The criteria to keep a variable in the multivariate model was obtaining a p < 0.05, and for its exclusion a p > 0.10. To calculate the effect size the coefficient of multiple correlation, R, was used. To measure the percentage of the variance predicted by the multiple regression model, the corrected or adjusted R2 was used. According to Cohen [27], R2 can be classified as insignificant when it is <0.02; small if it is between 0.02 and 0.15; medium if it is between 0.15 and 0.35; large if > 0.35. The assumptions of the model were checked paying special attention to the possible presence of multi-collinearity, which was verified when the condition indices were greater than 10 [28].

Data handling and analysis were conducted using the statistical package for social sciences (SPSS) version 21 (SPSS Inc., Chicago, IL, USA). A level of confidence of 95% was used (p < 0.05). 

## 3. Results

Fifty-seven patients met the inclusion criteria and completed all the planned study tests. The sociodemographic and most relevant clinical factors of the study sample are presented in Table 1**.** The sample had a mean age of 55.56 years (SD = 7.97) and a mean BMI of 28.68 kg/m^2^ (SD = 5.26), indicating overweight. Ninety-three percent were women and seven percent men. No association was found between these characteristics of the sample and the study variables. 

The impact of fibromyalgia as measured with the FIQ questionnaire and had a mean total score of 68.69 (SD = 13.97); a moderately severe impact on quality of life. Balance confidence, measured with the ABC scale, obtained data showing moderate balance confidence levels, with a mean score of 58.05 (SD = 22.34). The majority of our sample (61.4%) obtained confidence scores lower than 67%, indicating a substantial risk for falls.

Participants reported a mean of 4.28 (SD = 2.16) days with episodes of instability in the preceding week, noting feelings of fear of movement with a moderately-high level on the TSK (X = 28.88, SD = 7.11), and a moderated degree of catastrophic thoughts on the PCS questionnaire (X = 27.37, SD = 11.05). On the other hand, the presence of high central sensitization was observed (X = 61.74, SD = 10.94) and a moderate disability related with a perceived severe sensation of vertigo reported in the DHI questionnaire (X = 53.71, SD = 20.81). 

The stabilometric measurements related with CoP displacements showed values particularly high during the recordings made with open eyes (OE) and even more so, as expected, with closed eyes (CE). (Table 2)

The functional tests that evaluate balance showed elevated percentages of positive results (pathological). The highest percentages of vestibular symptom response were obtained in the static balance test, the sensitized Romberg test and in the one-leg standing time tests; with values around 50% when the test was performed with open eyes and 90% when performed with closed eyes (Table 2).

In the rest of the dynamic balance and gait balance functional tests, equally high percentages of positive results (impaired balance) were obtained and exceeded 40% of the total sample (Table 2).

The bivariate analysis did not find an association between the FIQ and the ABC and these categorical variables. Table 3 shows the simple and multiple regression models. The correlational analysis showed a significant association between the FIQ and days with instability, central sensitization, catastrophism, kinesiophobia, and the severity of perceived vertigo. These same variables were those that were significantly associated with balance confidence (ABC). The multiple regression model for FIQ kept the variables central sensitization and the severity of perceived vertigo. With these two variables, the model predicted 56.6% of the variance for FIQ, therefore the corrected R2 can be described as high (adjusted R2 =0.566). The prediction model for balance confidence, measured with the ABC, kept the variables days with instability, kinesiophobia, and severity of perceived vertigo (Table 4). Using these three variables, the model explained 52.7% of the variance of the aforementioned variable (ABC), therefore, the corrected R2 can also be described as high (R2 adjusted = 0.527). The variable that was included in both prediction models was severity of perceived vertigo, as measured by the DHI.

## 4. Discussion

This study intended to analyze dynamic and static balance impairment in persons with FMS who present with a moderate level of impairment from the disease. Our results showed a frequency of impairment between 50% and 90% with tests performed with closed eyes, including the one-leg standing time test and other static balance tests, and a frequency higher than 40% of patients with impaired dynamic and gait balance. These data are consistent with other studies that also found a prevalence in these types of patients of around 50% [3,6]. In terms of our study’s main objective, the fibromyalgia impact score was found to be associated with the CSI and the DHI, which explained around 56% of the variance of the dependent variable—FIQ. With regards to balance confidence, days with episodes of instability, kinesiophobia, and DHI score, were the variables that also explained more than half of the variance of the ABC scale score.

The participants of this study were mostly women (93%) with a mean age of 55.56 years. The majority had a secondary level of education, were married or in a stable relationship, and actively employed at the time of data collection. The sociodemographic distribution of our sample was therefore similar to that of other studies with patients diagnosed with FMS. Nevertheless, no correlation was found between the sociodemographic data of the sample (age, BMI, civil status, education level, and occupation) and the study variables. 

One of the most important impairments of fibromyalgia is its impact on quality of life. In this study a FIQ > 50 was established as part of the inclusion criteria, resulting in a sample with a moderately–severe degree of impact on quality of life. The mean total score obtained with the FIQ was 68.508 (SD = 15.83), similar to those obtained in recent studies such as Correa-Rodríguez et al. [29], which reported a mean impact of 73.08 points (SD = 14.73) in a sample selected in the cities Jaen and Granada, and the study by Seto et al. [30] that found a mean of 58.3 points (SD = 18.8) in a patient population with FMS in Boston city. 

Self-confidence is considered a psychological characteristic and has received a lot of attention in the treatment of diverse chronic illnesses [31], among which is FMS. In the study by Eun-Young et al. [32], the ABC scale was identified as a significant predictor of falls. In this study it was found the majority of the study population had a moderate-low balance confidence level and reported a substantial risk of falls with a mean total score on the ABC scale of 58.05 (SD = 22.34), a score lower than that found by Jones et al. [3,8], (ABC = 73.23, SD = 24.02; ABC = 81.24, SD = 19.52) in two studies on balance and posture control in patients with FMS with disease impact averages of 59.26 (SD 17.85) and 54.06 (SD 17.75). This difference could be due to the higher disease impact values of the sample included in the present study at the time of data collection (FIQ = 68.69, SD = 13.97).

Fibromyalgia is associated with balance impairment and an increased frequency of falls [3]. No single mechanism exists that could explain the altered postural instability in patients with FMS, but it could be explained by vestibular, somatosensory, or postural reflex disturbances [33]. Along these lines, Meireles et al. [12], found that patients with FMS have a higher frequency of falls than patients with RA or healthy subjects. These data support prior reports that indicate the falls are common in persons with FMS. 

Recent studies [4,8,34], have found that persons with FMS have sensory deficits, measure with stabilometry, despite have a normal clinical neurological exam. In a study by Jones et al. [3], postural stability was a predictor of the severity of fibromyalgia as measured with the FIQ, indicating that the severity of FMS symptoms and a deficient physical function are also related to postural control and evidencing a significant correlation between balance confidence (measured with ABC) and disease severity measured with the FIQ. In line on that shown by Jones et al. [8] in another study that used stabilometry in fibromyalgia patients, our study sample presented high abnormal values and a notable difference in the stabilometric values recorded with open eyes compared with closed eyes. In this sense, RMSX stabilometric parameter has been found increased in patients at risk of falls [23].

According to Pérez-de-Heredia-Torres et al. [35] patients with fibromyalgia show poor balance and use different strategies to maintain an upright posture which could be associated with disorders of the vestibular system. Moreover, the balance deficits are associated with a negative impact on independence in daily life activities-. These results are comparable with those obtained in the present study in which the severity of vertigo, a factor which alters postural stability, was associated with the total FIQ score. Central sensitization, measured by CSI, and vertigo severity as measured by the DHI, explained 56.6% of the negative impact exerted by fibromyalgia on quality of life.

On the other hand, a strong association was found, significant and inverse, for days with episodes of instability, kinesiophobia, and the perceived severity of vertigo on the ABC scale, identifying them as predictive factors of the latter and explaining 52.7% of its variability. Balance confidence can be interpreted as the degree of trust with which patients perform specific and daily tasks without losing balance. 

This study has some limitations to consider. First, as it is a cross-sectional study of a correlational nature, we were unable to analyze and observe in a sequential manner the evolution of the study variables and thus obtain more conclusive data on the causal relationships between FMS and balance impairment, in addition to observing the process of change in any of the variables studied. Second, the small sample size, may have resulted in obtaining of data with less statistical significance for some variables of our study. However, due to the exploratory nature of the study, in terms of the high number of variables included in the analysis, very relevant information has been provided in the relationships between variables that had not previously been associated in the literature in this study population. The relevance of this study is based on the analysis of variables are predictors of the impact of FMS. Variables associated with confidence in balance are also analyzed. This study highlights that disability due to dizziness on daily life is significantly related to the impact of the disease and balance confidence. This is a previous step to introduce intervention programs for patients with FMS. Finally, this study delves into the already known relationship between impaired balance and fibromyalgia.

Based on these results, future studies should include larger FMS population with lower levels of FIQ to analyze whether this condition affects this association between variables, as well as new studies that analyze the efficacy of a vestibular rehabilitation program to the impact of FMS.

## 5. Conclusions

The disease severity (measured with FIQ) in patients with a moderate-severe impact of FMS was associated with central sensitization (CSI) and the impact of dizziness on daily life (DHI), which explained 56% of its variance, while days with episodes of instability, kinesiophobia (TSK) and dizziness on daily life (DHI) also explained more than half of the variance of the balance confidence (measured with ABC Scale). 

A high frequency of positive results in functional balance tests and a high variability in the scores for stabilometric variables were found. 

The detection of factors susceptible to intervention, such as disability due to dizziness and vertigo, takes on special relevance in patients with FMS as it is significantly related to the impact of the disease and balance confidence.

## Figures and Tables

**Table 1:** Descriptive characteristics of patients with moderate-severe impact of fibromyalgia syndrome (FMS).

Variables	Cases (%)	Mean (SD)
Gender		
Female	53 (93)	
Male	4 (7)	
Age (years)		55.56 (7.97)
BMI (kg/m^2^)		28.68 (5.26)
Marital Status		
Single	4 (7)	
Divorced	9 (15.8)	
Married	43 (75.4)	
Widowed	1 (1.8)	
Education level		
None	1 (1.8)	
Primary	17 (29.8)	
Secondary	24 (42.1)	
University	15 (26.3)	
Employment status		
Professional active	28 (49.1)	
Sick leave	10 (17.5)	
Unemployment	10 (17.5)	
Retired	9 (15.8)	
Dependent variables:		
FIQ (0–100)		68.69 (13.97)
ABC (0–100)		58.05 (22.34)
Predictor variables:		
Days with instability (0–7)		4.28 (2.16)
CSI (0–100)		61.74 (10.94)
PCS (0–52)		27.37 (11.05)
TSK (11–44)		28.88 (7.11)
DHI (0–100)		53.71 (20.81)

ABC: activities-specific balance confidence scale; BMI: body mass index; CSI: central sensitization inventory; DHI: dizziness handicap inventory; FIQ: fibromyalgia impact questionnaire; PCS: pain catastrophizing scale; TSK: Tampa scale for kinesiophobia.

**Table 2:** Stabilometric parameters and balance tests of patients with moderate-severe impact of FMS.

Stabilometric Parameters:	Open/Closed Eyes	Mean (SD)	
Speed (mm/sec)	OE	19.20 (5.11)	
	CE	22.93 (5.80)	
RMSX (mm)	OE	0.35 (0.09)	
	CE	0.42 (0.12)	
RMSY (mm)	OE	0.41 (0.13)	
	CE	0.52 (0.23)	
X mean (mm)	OE	−2.66 (5.98)	
	CE	−3.17 (6.54)	
Y mean (mm)	OE	−18.93 (10.63)	
	CE	−20.70 (11.96)	
SVV (−2.5; 2.5)		−1.25 (2.99)	
Balance Tests:		Cases (%)	
		Positive	Negative
Romberg	OE	3 (5.3)	54 (94.7)
	CE	24 (42.1)	33 (57.9)
Sensitized Romberg l	OE	32 (56.1)	25 (43.9)
	CE	54 (94.7)	3 (5.3)
Sensitized Romberg r	OE	28 (49.1)	29 (50.9)
	CE	49 (86)	8 (14)
One-leg standing time l	OE	33 (57.9)	24 (42.1)
	CE	53 (93)	4 (7)
One-leg standing time r	OE	30 (52.6)	27 (47.4)
	CE	53 (93)	4 (7)
Fukuda-Unterberger test	OE	3 (5.3)	54 (94.7)
	CE	29 (50.9)	28 (49.1)
Fukuda left cervical rotation	CE	23 (40.4)	34 (59.6)
Fukuda right cervical rotation	CE	25 (43.9)	32 (56.1)
Babinski-Weill test		31 (54.4)	26 (45.6)
Tandem Walk test		23 (40.4)	34 (59.6)

CE: closed eyes; OE: open eyes; one-leg standing time l: left leg stance test; one-leg standing time r: right leg stance test; RMSX: root mean squared amplitude of the center of pressure in mediolateral direction; RMSY: root mean squared amplitude of the center of pressure in antero-posterior direction; sensitized Romberg l: with left leg back. Sensitized Romberg r: with right leg back; speed: speed of center of pressure; SVV: subjective visual vertical test; X mean: mean position of the center of pressure in the mediolateral plane; Y mean: mean position of the center of pressure in the antero-posterior plane.

**Table 3:** Simple and multiple linear regression model to predict FIQ.

Variables	Simple Regression		Multiple Regression	
	(r)	*p*-Value	Adjusted R^2^	*p*-Value
Age (years)	0.010	0.941	-	-
BMI (kg/m^2^)	0.029	0.829	-	-
Days with instability	0.268	0.044	NS	NS
CSI	0.615	0.001	0.384	0.001
PCS	0.389	0.003	NS	NS
TSK	0.278	0.036	NS	NS
DHI	0.702	0.001	0.566	0.001
Speed (mm/sec)				
OE	0.011	0.934	-	-
CE	0.043	0.754	-	-
RMSX (mm)				
OE	−0.018	0.894	-	-
CE	0.020	0.887	-	-
RMSY (mm)				
OE	0.064	0.644	-	-
CE	−0.013	0.923	-	-
X mean (mm)				
OE	0.042	0.759	-	-
CE	0.022	0.872	-	-
Y mean (mm)				
OE	−0.011	0.934	-	-
CE	−0.019	0.899	-	-
SVV	0.193	0.150	-	-

ABC: activities-specific balance confidence scale; BMI: body mass index; CE: closed eyes; CSI: central sensitization inventory; DHI: dizziness handicap inventory; FIQ: fibromyalgia impact questionnaire; NS: no significance; OE: open eyes; PCS: pain catastrophizing scale; RMSX: root mean squared amplitude of the center of pressure in mediolateral direction; RMSY: root mean squared amplitude of the center of pressure in antero-posterior direction; speed: speed of center of pressure; SVV: subjective visual vertical test; TSK: Tampa scale for Kinesiophobia; X mean: mean position of the center of pressure in the mediolateral plane. Y mean: mean position of the center of pressure in the antero-posterior plane.

**Table 4:** Simple and multiple linear regression to predict ABC score.

Variables	Simple Regression		Multiple Regression	
	(r)	*p*-Value	Adjusted R^2^	*p*-Value
Age (years)	−0.126	0.349	-	-
BMI (kg/m^2^)	0.009	0.947	-	-
Days with instability	−0,620	0.001	0.373	0.003
CSI	−0.398	0.002	NS	NS
PCS	−0.317	0.016	NS	NS
TSK	−0.524	0.001	0.483	0.017
DHI	−0.600	0.001	0.527	0.002
Speed (mm/sec)				
OE	0.128	0.350	-	-
CE	0.158	0.249	-	-
RMSX (mm)				
OE	−0.071	0.606	-	-
CE	−0.014	0.920	-	-
RMSY (mm)				
OE	0.117	0.394	-	-
CE	0.072	0.601	-	-
X mean (mm)				
OE	0.092	0.504	-	-
CE	0.027	0.842	-	-
Y mean (mm)				
OE	0.090	0.515	-	-
CE	0.053	0.700	-	-
SVV	−0.073	0.587	-	-

ABC: activities-specific balance confidence scale; BMI: body mass index; CE: closed eyes; CSI: central sensitization inventory; DHI: dizziness handicap inventory; FIQ: fibromyalgia impact questionnaire; NS: no significance; OE: open eyes; PCS: pain catastrophizing scale; RMSX: root mean squared amplitude of the center of pressure in mediolateral direction; RMSY: root mean squared amplitude of the center of pressure in antero-posterior direction; speed: speed of center of pressure; SVV: subjective visual vertical test; TSK: Tampa scale for kinesiophobia; X mean: mean position of the center of pressure in the mediolateral plane. Y mean: mean position of the center of pressure in the antero-posterior plane.

## References

[1] Wolfe F., Clauw D.J., Fitzcharles M.A., Goldenberg D.L., Katz R.S., Mease P., Russell A.S., Russell I.J., Winfield J.B., Yunus M.B. (2010). The American College of Rheumatology preliminary diagnostic criteria for fibromyalgia and measurement of symptom severity. Arthritis Care Res..

[2] Mease P., Arnold L.M., Bennett R., Boonen A., Buskila D., Carville S., Chappell A., Choy E., Clauw D., Dadabhoy D. (2007). Fibromyalgia syndrome. J. Rheumatol..

[3] Jones K.D., Horak F.B., Winters K.S., Morea J.M., Bennett R.M. (2009). Fibromyalgia is associated with impaired balance and falls. J. Clin. Rheumatol..

[4] Muto L.H., Sauer J.F., Yuan S.L., Sousa A., Mango P.C., Marques A.P. (2015). Postural control and balance self-efficacy in women with fibromyalgia: Are there differences?. Eur. J. Phys. Rehabil. Med..

[5] Trevisan D.C., Driusso P., Avila M.A., Gramani-Say K., Moreira F.M.A., Parizotto N.A. (2017). Static postural sway of women with and without fibromyalgia syndrome: A cross-sectional study. Clin. Biomech..

[6] Bennett R.M., Jones J., Turk D.C., Russell I.J., Matallana L. (2007). An internet survey of 2,596 people with fibromyalgia. BMC Musculoskelet. Disord..

[7] Peterka R.J. (2003). Simplifying the complexities of maintaining balance. IEEE Eng. Med. Biol. Mag..

[8] Jones K.D., King L.A., Mist S.D., Bennett R.M., Horak F.B. (2011). Postural control deficits in people with fibromyalgia: A pilot study. Arthritis Res. Ther..

[9] Collado-Mateo D., Gallego-Diaz J.M., Adsuar J.C., Domínguez-Muñoz F.J., Olivares P.R., Gusi N. (2015). Fear of falling in women with fibromyalgia and its relation with number of falls and balance performance. Biomed. Res. Int..

[10] Rasouli O., Stensdotter A.K., Van der Meer A.L. (2016). TauG-guidance of dynamic balance control during gait initiation in patients with chronic fatigue syndrome and fibromyalgia. Clin. Biomech..

[11] Russek L.N., Fulk G.D. (2009). Pilot study assessing balance in women with fibromyalgia syndrome. Physiother. Theory Pract..

[12] Meireles S.A., Antero D.C., Kulczycki M.M., Skare T.L. (2014). Prevalence of falls in fibromyalgia patients. Acta Ortop. Bras..

[13] Rutledge D.N., Cherry B.J., Rose D.J., Rakovski C., Jones C.J. (2010). Do fall predictors in middle aged and older adults predict fall status in persons 50+ with fibromyalgia? An exploratory study. Res. Nurs. Health.

[14] Monterde S., Salvat I., Montull S., Fernández-Ballart J. (2004). Validación de la versión española del Fibromyalgia Impact Questionnaire. Rev. Esp. Reumatol..

[15] Montilla-Ibáñez A., Martínez-Amat A., Lomas-Vega R., Cruz-Díaz D., Torre-Cruz M.J.D.L., Casuso-Pérez R., Hita-Contreras F. (2017). The Activities-specific Balance Confidence scale: Reliability and validity in Spanish patients with vestibular disorders. Disabil. Rehabil..

[16] Horak F.B. (2006). Postural orientation and equilibrium: What do we need to know about neural control of balance to prevent falls?. Age Ageing.

[17] Cuesta-Vargas A.I., Roldan-Jimenez C., Neblett R., Gatchel R.J. (2016). Cross-cultural adaptation and validity of the Spanish central sensitization inventory. Springerplus.

[18] García-Campayo J., Rodero B., Alda M., Sobradiel N., Montero J., Moreno S. (2008). Validación de la versión española de la escala de la catastrofización ante el dolor (Pain Catastrophizing Scale) en la fibromialgia. Med. Clin..

[19] Gómez-Pérez L., López-Martínez A.E., Ruiz-Párraga G.T. (2011). Psychometric properties of the Spanish version of the Tampa Scale for Kinesiophobia (TSK). J. Pain..

[20] Pérez N., Garmendia I., Martin E., Garcia-Tapia R. (2000). Adaptación cultural de dos cuestionarios de medida de salud en pacientes con vértigo. Acta Otorrinolaringol. Esp..

[21] Negrillo-Cárdenas J., Rueda-Ruiz A.J., Ogayar-Anguita C.J., Lomas-Vega R., Segura-Sánchez R.J. (2018). A System for the Measurement of the Subjective Visual Vertical using a Virtual Reality Device. J. Med. Syst..

[22] Piscicelli C., Pérennou D. (2017). Visual verticality perception after stroke: A systematic review of methodological approaches and suggestions for standardization. Ann. Rehabil. Med..

[23] Hita-Contreras F., Martínez-Amat A., Lomas-Vega R., Álvarez P., Aránega A., Martínez-López E., Mendoza N. (2013). Predictive value of stabilometry and fear of falling on falls in postmenopausal women. Climacteric.

[24] Cohen H.S. (2019). A review on screening test for vestibular disorders. J. Neurophysiol..

[25] Seichi A., Hoshino Y., Doi T., Akai M., Tobimatsu Y., Kita K., Iwaya T. (2014). Determination of the optimal cutoff time to use time to screening elderly people for locomotive syndrome using the one-leg standing test (with open eyes). J. Orthop. Sci..

[26] Paquet N., Jehu D.A., Lajoie Y. (2017). Aged-related differences in Fukuda stepping test and Babinski Weil test, within-day variability and test-retest reliability. Aging Clin. Exp. Res..

[27] Cohen J. (1992). A power primer. Psychol. Bull..

[28] Yu H., Jiang S., Land K.C. (2015). Multicollinearity in hierarchical linear models. Soc. Sci. Res..

[29] Correa-Rodríguez M., El Mansouri-Yachou J., Casas-Barragán A., Molina F., Rueda-Medina B., Aguilar-Ferrándiz M.E. (2019). The Association of Body Mass Index and Body Composition with Pain, Disease Activity, Fatigue, Sleep and Anxiety in Women with Fibromyalgia. Nutrients.

[30] Seto A., Han X., Price L.L., Harvey W.F., Bannuru R.R., Wang C. (2019). The role of personality in patients with fibromyalgia. Clin. Rheumatol..

[31] Jones F., Riazi A. (2011). Self-efficacy and self-management after stroke: A systematic review. Disabil. Rehabil..

[32] Eun-Young Park P.T., Young-Jung Lee P.T., Yoo-Im Choi O.T. (2018). The sensitivity and specificity of the Falls Efficacy Scale and the Activities-specific Balance Confidence Scale for hemiplegic stroke patients. J. Phys. Ther. Sci..

[33] Akkaya N., Akkaya S., Atalay N.S., Acar M., Catalbas N., Sahin F. (2013). Assessment of the relationship between postural stability and sleep quality in patients with fibromyalgia. Clin. Rheumatol..

[34] Yang J.F., Winter D.A., Wells R.P. (1990). Postural dynamics in the standing human. Biol. Cybern..

[35] Pérez-de-Heredia-Torres M., Huertas-Hoyas E., Martínez-Piédrola R., Palacios-Ceña D., Alegre-Ayala J., Santamaría-Vázquez M., Fernández-de-las-Peñas C. (2017). Balance deficiencies in women with fibromyalgia assessed using computerised dynamic posturography: A cross-sectional study in Spain. BMJ Open.

